# Diagnostic accuracy and feasibility of a rapid SARS-CoV-2 antigen test in general practice – a prospective multicenter validation and implementation study

**DOI:** 10.1186/s12875-022-01756-1

**Published:** 2022-06-11

**Authors:** Jörn Rohde, Wolfgang Himmel, Clemens Hofinger, Thiên-Trí Lâm, Hanna Schrader, Julia Wallstabe, Oliver Kurzai, Ildikó Gágyor

**Affiliations:** 1 Department of General Practice, Julius-Maximilians-Universität Wuerzburg, Wuerzburg, Germany; 2 Department of General Practice, University Medical Center Goettingen, Goettingen, Germany; 3 Institute for Hygiene and Microbiology, Julius-Maximilians University of Wuerzburg, Wuerzburg, Germany; 4 Leibniz Institute for Natural Product Research and Infection Biology, Hans-Knoell-Institute, Jena, Germany

**Keywords:** COVID-19 testing, General practice, Sensitivity and specificity, Attitude of health personnel, Feasibility study

## Abstract

**Background:**

PCR testing is considered the gold standard for SARS-CoV-2 diagnosis but its results are earliest available hours to days after testing. Rapid antigen tests represent a diagnostic tool enabling testing at the point of care. Rapid antigen tests have mostly been validated by the manufacturer or in controlled laboratory settings only. External validation at the point of care, particularly in general practice where the test is frequently used, is needed. Furthermore, it is unclear how well point of care tests are accepted by the practice staff.

**Methods:**

In this prospective multicenter validation study in primary care, general practitioners included adult individuals presenting with symptoms suggesting COVID-19. Each patient was tested by the general practitioner, first with a nasopharyngeal swab for the point of care test (Roche SARS-CoV-2 Rapid Antigen Test) and then with a second swab for PCR testing. Using the RT-PCR result as a reference, we calculated specificity, sensitivity, positive predictive value and negative predictive value, with their 95% confidence intervals. General practitioners and medical assistants completed a survey to assess feasibility and usefulness of the point of care tests.

**Results:**

In 40 practices in Würzburg, Germany, 1518 patients were recruited between 12/2020 and 06/2021. The point of care test achieved a sensitivity of 78.3% and a specificity of 99.5% compared to RT-PCR. With a prevalence of 9.5%, the positive predictive value was 93.9% and the negative predictive value was 97.8%. General practitioners rated the point of care test as a helpful tool to support diagnostics in patients with signs and symptoms suggestive for infection, particularly in situations where decision on further care is needed at short notice.

**Conclusion:**

The point of care test used in this study showed a sensitivity below the manufacturer’s specification (Sensitivity 96.25%) in the practice but high values for specificity and high positive predictive value and negative predictive value. Although widely accepted in the practice, measures for further patient management require a sensitive interpretation of the point of care test results.

**Supplementary Information:**

The online version contains supplementary material available at 10.1186/s12875-022-01756-1.

## Introduction

Reverse transcription polymerase chain reaction (RT-PCR) tests are considered the gold standard for the diagnosis of SARS-CoV-2 infections [1, 2]. However, they have some limitations e.g. a long turnaround time reaching up to 24 hours or more, high demands regarding infrastructure, and relatively high costs [3–5]. Suitable alternatives particularly for the use in primary care could be rapid antigen tests as already demonstrated, e.g. for the diagnosis of streptococcal tonsillitis or influenza [6–8]. These tests should be characterized by rapid and cost-effective results, user-friendly handling, and low infrastructural requirements [9]. The pandemic has increased the overall workload of general practitioners (GPs) [10]. In this context, it is important to investigate how well a rapid test can actually get integrated into the already stressful workday.

Rapid tests must have a sensitivity of at least 80% and a specificity of at least 97% for approval in Germany by the Federal Institute for Drugs and Medical Devices (BfArM) [11]. Although the Paul Ehrlich Institute demonstrated high sensitivity for many commercial test, most of them were validated by the manufacturer or in controlled laboratory settings only. However, external validation at the point of care, where the test is frequently used, is needed. GPs play a key role in the pandemic as they are usually the first contact for patients. About 34% of corona cases identified in the first half of 2020 were treated by the GPs [12]. In addition to patient care involving diagnosis, treatment and patient education, general practice is determined to be a place to go for testing [13, 14].

The validation studies of the manufacturers often include only small study samples with a very high prevalence. These study populations are not comparable to real world health care. While there are independent validations performed under controlled laboratory conditions that already provide valuable information, several studies were conducted in primary care settings such as test centers [15–18]. However, the settings in test centers are far away from the typical routines and requirements of a GP practice. Only a few studies were conducted in primary healthcare centers and it is also unclear, whether the patient population is that of a general practice [19, 20].. We only found one study that was actually conducted in general practices [21]. As most patients with a COVID19 have been treated in general practice, this setting seemed underreported and most suitable to conduct the study. Validation data and feasibility studies directly from general practices are, to our knowledge, not yet available in sufficient numbers and are urgently needed.

The aims of this study were twofold: [1] to evaluate the diagnostic accuracy of a SARS-CoV-2 rapid antigen test in primary care and [2] to investigate how well the test is accepted by the practice staff.

## Material and methods

### Study design

This prospective multicenter validation study in general practice was conducted in 40 general practices in Würzburg, a city of nearly 130,000 inhabitants in the federal state of Bavaria, and its surrounding area from December 2020 to June 2021. During this period, the predominant virus variant was the alpha variant. From May 2021, it was largely replaced by the delta variant [22].

### Study population

Inclusion criteria for patients were based on the PCR test guidelines of the Robert Koch Institute [23], the national public health authority in Germany, which, besides others, is continuously monitoring the COVID-19 situation and estimating the risk for the population. We included adult patients with any acute, recently appeared respiratory symptoms and/or symptoms suggesting a SARS-CoV-2 infection such as smell and/or taste disorders (hypo- or anosmia, hypo- or ageusia) [23]. Participating GPs should check patients for eligibility and include them consecutively in the study after written consent. Patients should complete a clinical questionnaire asking for age, sex, contact with a SARS-CoV-2 positive person, symptoms (see Tab. 1) and days since symptom onset.

**Table 1:** Description of study population

	Prevalence, n: 1.450*	
Variables	Test pos.; % (n)	Test neg.; % (n)
n	138	1312
Age; m^a^	43	40
Contact to a person with COVID-19, *N* = 107	34.1 (47)	5.1 (60)
Gender		
Female	45.7 (59)	46.3 (578)
Male	54.3 (70)	53.7 (671)
Days since symptom onset		
Day 1–3	66.7 (84)	73.4 (886)
Day 3–7	27 (34)	20.6 (249)
Day > 7	6.3 (8)	6 (72)

*missing values range between 81 and 136

^a^Median

### Test procedure

The GPs were responsible for maintaining hygiene standards during their consultations. Most GPs performed the point of care test (POCT) as part of a special infectious disease consultation. GP took two nasopharyngeal swabs from each patient. The first swab for POCT and the second swab for PCR testing. According to the manufacturer’s protocol of the Roche SARS-CoV-2 Rapid Antigen Test [24], the practice staff read the result of the antigen test after 15–30 minutes. If the test showed a clear positive result earlier than 15 minutes, this positive test result was considered as valid. The second swab for RT-PCR was sent to one of six different cooperating laboratories. All laboratories were certified according to DIN EN ISO 15189:2014 or ISO 9001:2015 [25, 26]. If one of the two tests (POCT or RT-PCR) was positive, an antibody (Ab) serology was offered on a voluntary basis in order to better assess the clinical case by adding an additional test method [27]. The blood sample was taken at the respective GP office earliest 10 days after positive test result. The analysis was conducted at the Institute for Hygiene and Microbiology, Würzburg. All test results and the symptom questionnaire were linked by a study ID.

### Sample size calculation and statistical analysis

For the evaluation of the antigen test compared to PCR results, a 95% confidence interval of +/− 5% for sensitivity and specificity was considered adequate. Assuming a prevalence of 9%, a sensitivity of 78% and a specificity of 99%, the required sample size was 2930 participants.

Baseline characteristics of patients were analyzed descriptively and were expressed as percent, median with interquartile range (IQR) or mean with standard deviation (SD). To test for differences between groups, Fischer’s exact two-tailed tests were performed in case of nominal variables and the Mann-Whitney U test in case of metric and categorical variables.

To determine the accuracy of the antigen tests, we calculated specificity, sensitivity, positive and negative predictive value (PPV, NPV), with their 95% confidence intervals (95% CI). The values were calculated using the RT-PCR result as a reference. We broke down the cycle threshold (Ct-) values as a proxy for the viral load into groups and determined the sensitivity for each group. We split the PCR-positive results into two groups. In one group, PCR and POCT results matched (true positive), in the other the POCT falsely showed a negative result (false negative). The difference between the two groups was plotted as a function of the Ct value to determine the specific difference.

### Survey of medical staff

GPs and medical assistants (MAs) who were involved in the organization, implementation and execution of the rapid antigen test were interviewed using a standardized questionnaire about the feasibility and acceptance of the procedure at the end of the study. After eliciting some baseline characteristics, GPs and assistants could rate the organizational and logistical effort of the test procedure as well as consequences for medical treatment on six-point Likert scales, for example ranging from very good to very bad or not useful to very useful. In addition, we encouraged participants to provide free text responses in order to achieve a higher data saturation (for the detailed questionnaire, see attachment). For the conduct of the survey we used the survey software EvaSys [28].

Data analyses were performed using IBM SPSS Statistics (Version 26) software [29].

## Results

### Study population

**Fig. 1:**
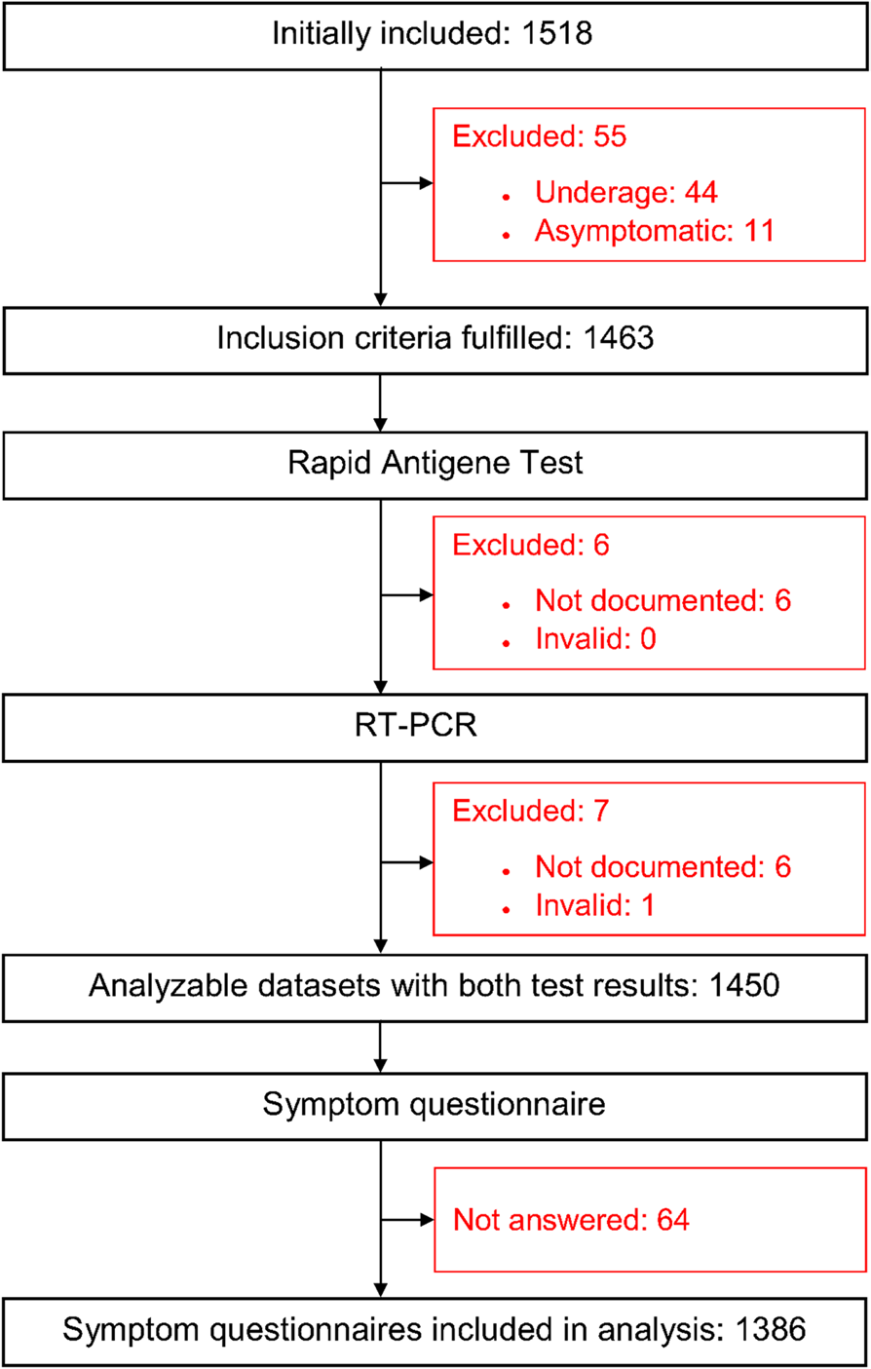
Flow of study participants: Initially included participants with reasons for exclusion

During the first 4 months of the recruitment period (until April 2021), we could enroll between 144 and 615 patients per month. In May and June, it was only possible to recruit 72 and 2 patients, respectively. Therefore, we decided to stop recruitment. The participating 40 general practices recruited 1518 patients, on average, 36.3 (SD 27; Range: 3 to 141) patients per practice (Fig. 1). After exclusion of 68 patients (0.04%), because they did not meet inclusion criteria, the final sample consisted of 1450 patients, 1386 (95.6%) of them partly or fully completed the symptom questionnaire.

Of the included patients, 730 of 1450 (53%) were female; median age was 40 years (IQR 30 to 55). Most patients (1253/1450 ≙ 86%) consulted their GP within the first 7 days after symptom onset (Table 1). On median, the initial consultation and testing took place 2 days (IQR 1.5 to 4) after symptom onset.

### Diagnostic accuracy of the rapid antigen test

Of the patients, 138 of 1450 (9.5%) had a PCR-positive test. Of the patients, 138 of 1450 (9.5%) had a PCR-positive test. Of these, 108 also had a positive antigen test result yielding a sensitivity of 78.3%, 30 patients had negative antigen test results (considered as false negative result). Seven of 1312 patients with PCR negative test results had positive rapid antigen test results (considered false positive), yielding a specificity of 99.5%. In our study population with a SARS-CoV-2 prevalence of 9.5%, the rapid test achieved a PPV of 93.9% and an NPV of 97.8% (Table 2).

**Table 2:** Overview of the results: Comparison of the rapid antigen test and PCR for SARS-CoV-2

		RT-PCR		
		Positiv	Negativ	Total
Rapid antigen test	Positiv	108	7	115
	Negativ	30	1305	1335
	Total	138	1312	1450
		%	(95% CI)^a^	
Prevalence		9.5		
Specificity		99.5	(99.0–99.8)	
Sensitivity		78.3	(70.9–84.6)	
NPV		97.8	(96.9–98.5)	
PPV		93.9	(88.6–97.3)	
^a^95% CI 95% confidence intervall.				

**Fig. 2:**
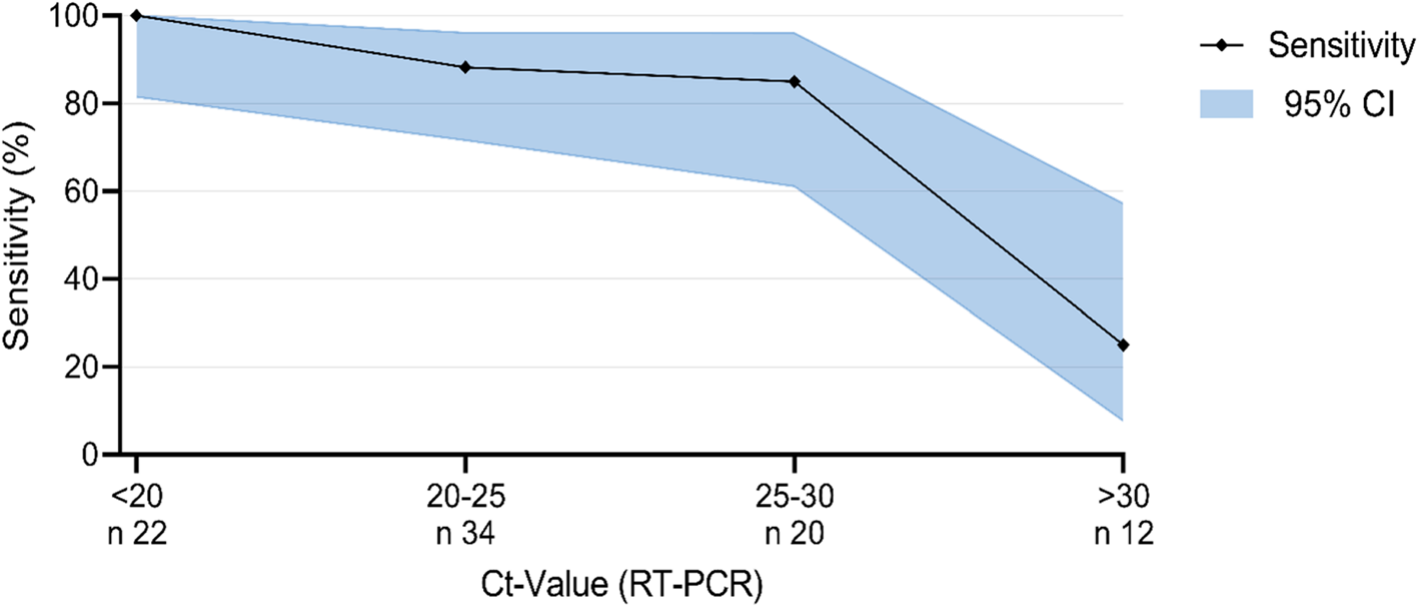
Viral concentration (Ct-value) of true positive rapid antigen tests (*n* = 72) versus false negative tests (*n* = 16)

**Fig. 3:**
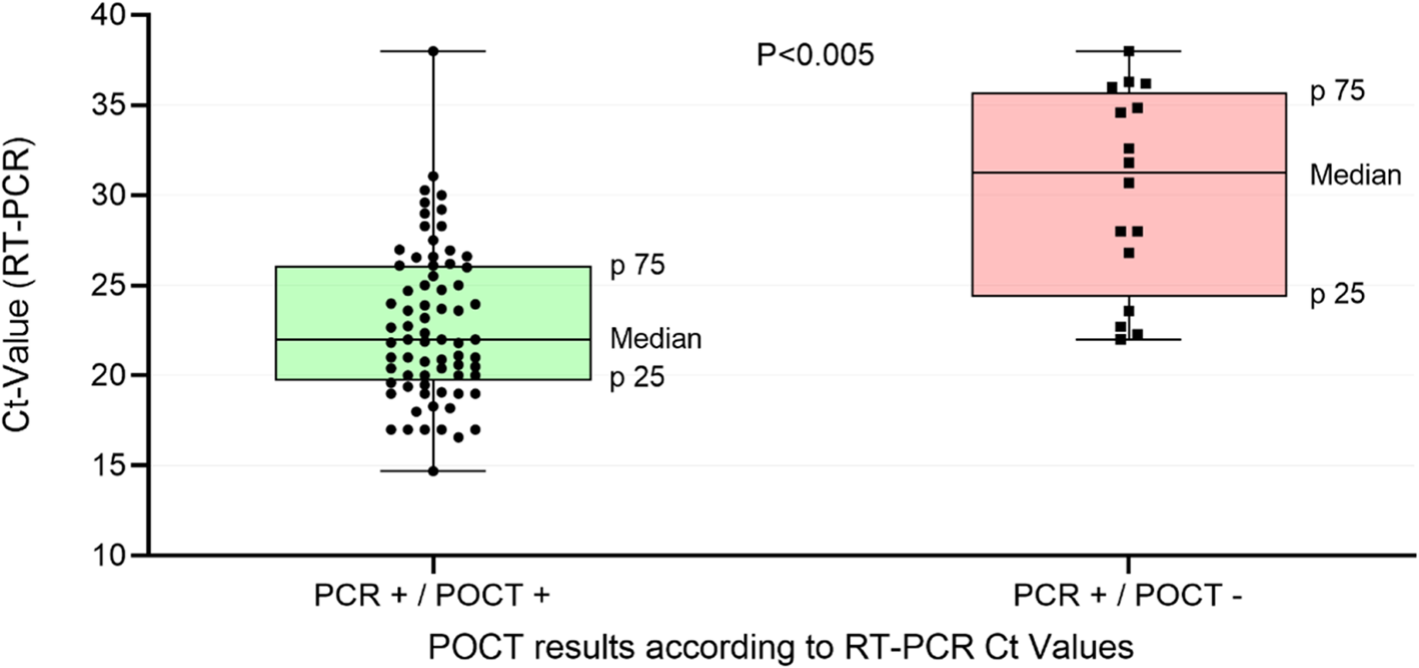
Sensitivity of the rapid antigen test according to CT-values (*n* = 88)

We investigated the sensitivity in relation to the Ct-value. The median Ct-value was 23 (IQR 20.3 to 27). The detection rate decreased significantly with increasing (Ct-value i.e. decreasing viral load). For example, the sensitivity for patients with a Ct-value of > 30 was only 25% (*n* = 12; 95% CI: 7.7–57.2%), whereas for a Ct value of ≤30 the sensitivity was 90.8% (*n* = 69; 95% CI: 81.4–95.9%) (Fig. 2). We were able show that the Ct values for true positive results were significantly lower than for false negative results (*p*-value: < 0.005). Figure 3 shows the dispersion of the individual test results (true positive, false negative) over the Ct-value scale.

### Serological testing

The opportunity to determine seroconversion was offered to 145 patients who tested positive, 51 (35.2%) accepted the offer.

We defined seven cases as “false positive” (Table 2). Of the seven patients, we performed a serology in three cases. Against expectations, all of them showed a clear positive seroconversion. Independently of this study, we received reports from the GPs that two of them underwent further RT-PCR which led to positive results.

Furthermore, we defined 30 cases as “false negative” (Tab. 2). Of these, we performed serology in eleven cases, only six cases showed seroconversion with positive Ab-titers. Five cases showed a negative Ab-titer.

Moreover, we performed serology in 37 cases defined as “true positive”. Here, 35 cases showed a clearly positive seroconversion.

### Acceptance of the test by the practice staff

A total of 40 GPs and 39 MA completed the survey to evaluate the acceptance of the POCT. Almost half of the GPs were female (48.6%), 46% of them were between 45 and 54 years old and 61% worked in a group practice. The assistants were mostly female (92%), 58% of them were between 35 and 54 years old and they were predominantly (62%) active in a group practice. For more detailed information, see Table 1 in the attachment.

#### Feasibility of the test procedure

The participants estimated that pure working time for the execution of a test was, in median, 5 (IQR 4) minutes. Overall, the GPs rated the feasibility as easy, with a median score of 2 (*n* = 39, IQR 1 to 2) on a scale from 1 (very simple) to 6 (very complicated). The MAs perceived the feasibility of the tests in everyday practice as moderate, with a median score of 3 (*n* = 39, IQR 2 to 3) on a scale of 1 (very good) to 6 (very bad). In the free text section of this question, MAs reported the increased documentation work as a burden.

#### Perceived benefits of the antigen test

GPs and MAs largely agreed that the use of rapid antigen testing could have a positive impact on infection control. On a scale from 1 (strongly agree) to 6 (strongly disagree), GPs rated the statement with 4.5 (SD1.5) and MAs with 4.5 (SD1.4). Nearly all of the GPs (92.1%) and assistants (94.6%) agreed that the tests performed by the GPs were superior to lay test (over-the-counter tests). GPs reported that the antigen test helped them in clinical decision making in more than half of the tested patients (5.3/10, SD 3.9). Consequences of the antigen test results were, for example, an earlier initiated quarantine, rapid risk assessment and early close-meshed care (frequent telephone contact, monitoring of the health status, in time hospitalization if necessary) in case of a positive result (see Table 3). Almost two thirds (64.1%, *n* = 39) of GPs even stated that there were situations in which they would prefer to use rapid antigen tests in the first place. Particularly in situations in which rapid decision-making is necessary, for example, in symptomatic high-risk patients or emergency cases that need to be admitted to the hospital (Table 3).

**Table 3:** Representative citations to the free text sections of the survey. (Original language German)

Situations in which the POCT was a help for further medical treatment	
GP	“Rapid statement also in the context of contact tracing as the PCR test often takes a long time. One patient tested positive in a POCT prior to the weekend ➔ close-meshed telephone “monitoring” then still in time hospitalization”.
GP	“Positive rapid test with corresponding symptoms clearly indicates suspicion of COVID. As a consequence, quarantine measures and notification to health department have already been possible prior to the weekend.”
Situations in which the POCT is preferred.	
GP	“Home visit, decision on hospital admission, high-risk individuals.”
GP	“If symptoms are severe: high fever, dyspnea, reduced general health, family accumulation of disease, preexisting diseases such as COPD or asthma, or immunosuppressed patients”
GP	“Before visiting sick person if PCR test result would take too long.”

Original language German

## Discussion

Given the common use of rapid testing in GP practices, data on the validity of this testing strategy at this particular “point of care” are needed as well as knowledge about whether these tests are accepted by the practice staff and can easily be used in practice. With a sensitivity of 78.3% and a specificity of 99.5% compared to RT-PCR-tests, rapid testing in primary care practice yields comparably valid results as in controlled laboratory settings. Most GPs and MAs perceived rapid tests as easy to integrate into the daily routine of primary care and would use a POCT especially in situations with an immediate need for action. Since the sensitivity of POCT decreased strongly with increasing Ct-values, i.e., decreasing viral load, sufficient detection of the disease seems especially possible in acute stages of the disease with a high viral load (low Ct-value). Both tests, even RT-PCR-tests do not always provide error-free results.

For the Roche SARS-CoV-2 Rapid Antigen Test we used in this study we could show a very high specificity, similar to the manufacturer specifications. The measured sensitivity of 78% was far lower than the manufacturer’s reported sensitivity of 96.25% [24]. The Paul Ehrlich Insitute (PEI - German Federal Institute for Vaccines and Biomedicines) determined a sensitivity of only 30.4% for the Ct value of 25–30, but for a Ct value of ≤25, the sensitivity was still 88.9% [30]. As shown by the PEI data, we were able to prove that the sensitivity decreased with increasing Ct-values, i.e. decreasing viral load. GPs and medical assistants found the POCT easy to implement into everyday practice. Even though its performance is lower than a PCR test, many GPs would use a rapid antigen test especially in situations with an immediate need for action.

### Performance of the rapid antigen test

Professionally conducted antigen tests do not require manufacturer-independent validation to be approved for the German market [31]. When investigated in independent clinical studies, the sensitivities of the Roche SARS-CoV-2 Rapid Antigen Test in symptomatic patients ranged from less than 63% to well over 85% [15, 16, 32, 33]. The different results can probably be explained by different study characteristics (test site, inclusion criteria, staff training, etc.). Therefore, external manufacturer-independent validation in different clinical settings is needed. This allows diagnostic accuracy and scope of a test result to be interpreted correctly.

Like other studies before, our results show that the test provides reliable results especially at very low Ct-values, i.e. at a high viral load [34]. The viral load is dependent on the days since symptom onset. At the beginning of the infection, the viral load is particularly high and decreases over time [15, 35]. The Ct-value alone is not sufficient to exclude risk of transmission. Infectivity is significantly dependent on SARS-CoV-2 gene copy number as well as genome integrity [36]. However, the Ct-value correlates strongly with the viral load and for this reason, it can be a valuable tool for decision making and risk assessment [37]. Above a Ct-value of > 30, virus cultivation is difficult and infectivity seems unlikely [38]. We showed that the POCT does not always provide reliable results even at a Ct-value of less than 30 (Fig. 3), a range of diagnostic inaccuracy prevails especially at Ct-values between 20 and 30.

Our results showed a sufficiently high specificity of antigen tests in primary care practices. However, even if positive test results can be interpreted with high probability as true positives, they should be confirmed by a RT-PCR. In contrast, the sensitivity of the test was only moderate and strongly depended on the viral load. Rapid antigen tests cannot provide information about the viral load, and thresholds to rule out contagiousness are difficult to define. Furthermore, the test cannot distinguish between a beginning and a declining infection. If a patient is at the beginning of the infection, the viral load may have increased after a short time and infection of other people cannot be ruled out. A negative test result cannot exclude a transmission-relevant infection with certainty and should not lead to a false sense of security [39].

The high mutation potential of the coronavirus is also a recurring topic of discussion. The mutations mostly affect the S-protein. Rapid tests, on the other hand, usually detect the N-protein. Thus, the tests should still be able to detect infection even in the presence of mutations [40]. In the case of since recently dominating the omicron variant, however, initial data show that the sensitivity of the rapid test could be lower [41]. Regular re-evaluations seems absolutely necessary to detect diagnostic deficiencies in time.

It should be emphasized that the validity of the test strongly depends on the PPV, NPV and prevalence, respectively the pre-test probability. These values are highly dynamic due to the fluctuating character of the pandemic. Assuming that the PCR test is almost always correct, the prevalence among PCR-tested persons is approximately equal to the test positive rate. An example from reality; the test positive rate temporarily dropped to 0.82% during the summer in Germany [42]. For the rapid test, this would result in a calculated PPV of 56%. This implies, that almost half of all positive tests would be false-positive. In November 2021, the test positive rate reached up to 21.2% [43]. Here, 98% of the persons with a positive test would actually be infected. However, with a calculated NPV of 94.4%, over 5% of negative tested persons would have an active infection and could potentially pass on the virus.

In summary, the lower the prevalence, the less reliable are positive results, and the higher the prevalence, the less reliable are negative results [44]. To exclude infection, a POCT is best when the incidence of covid-19 is low, whereas in covid-19 waves, a negative antigen test should be followed by a PCR test to truly exclude covid-19 infection.

### Feasibility and benefits of the rapid antigen test

The additional test caused only minor logistical challenges for GPs and MA. Its regular implementation in everyday practice should be easily possible. GPs and MA considered a physician-performed test to be superior to lay testing because it is presumed to be more accurately administered. Vice versa, the public also seems to have the highest trust in physician-performed antigen tests. According to a survey in Germany, general practices are the preferred testing location for 33% of patients, pharmacies for 25% and testing centers for only 12% [14].

GPs considered positive test results helpful as they were perceived as very safe due to their high specificity and appropriate measures such as quarantine and specific medical care could be initiated immediately.

### Strengths and weaknesses

For the evaluation of the POCT, we used the PCR test as the gold standard. The PCR test is currently the most accurate way to detect an infection. However, this test does not always provide 100% certainty. This can lead to a slight bias in the results. Our RT-PCR samples were evaluated at different accredited laboratories. Despite of a consistently high quality standard, the Ct-values are only comparable to a limited extent. However, it can be assumed that the trends (high Ct-value = low viral load, low Ct-value = high viral load) are still correct. Furthermore, we could not consider the Ct-values of all positive patients because in some cases they were not documented by the practice or provided by the laboratories.

Two different nasopharyngeal swabs were taken consecutively from each patient. Differences in the two sample collections cannot be excluded.

MAs rated the feasibility of the tests worse than the GPs. In some cases, the additional documentation, which was needed for the study, was stated as a reason. In everyday practice without this study-specific documentation, the feasibility should achieve better ratings.

Various authors pointed out that antibody testing may serve as a complementary method for COVID-19 diagnostics [27, 45, 46]. To further verify the results of the POCT and especially PCR testing, we offered antibody testing to all positive tested participants. Unfortunately, only one third of the patients took up this voluntary offer. Another limitation of the antibody testing consisted in the fact that the study was carried out during the second major wave of Covid-19 in Germany. Even though reinfections were rare at the time, they cannot be ruled out upon positive antibody detection. However, although the performed serologies were able to confirm an infection in most of the cases, they also lead to the suggestion that the tests, including the RT-PCR test, do not always provide error-free results. Probably, a second additional PCR test could provide more reliability in future studies.

We hoped to reach the targeted sample size before summer when a strongly decreasing demand for POCT could be expected. As we realized that patient recruitment sharply decreased already in May, we decided to stop the recruitment phase, considering the comparably large group of participants, but had to accept an increase in confidence intervals, especially for sensitivity.

## Conclusions

A rapid antigen test is a feasible diagnostic tool for the detection of a SARS-CoV-2 infection in general practice. It showed a sensitivity below the manufacturer’s specification in the practice but high values for specificity and high PPV and NPV. Given these values, implementation requires a sensitive interpretation of the results in order to derive measures for further treatment. The POCT provides reliable results in the acute phase of the disease (with high viral load; Ct-value < 30). Nevertheless, GPs should be aware of false negative test results as a possible diagnostic gap especially at the beginning and end of the disease (at low viral load).

## Supplementary Information

**Additional file 1.** Questionnaire MA.

**Additional file 2.** Questionnaire GP.

**Additional file 3.** Attachement Table 1 Baseline characteristics of MAs ans GPs who followed the survey.

## Data Availability

The datasets generated and analyzed during this study are not publicly available due to further evaluation but are available from the corresponding author on reasonable request.

## Abbreviations

Ab: Antibody
CI: Confidence interval
COVID-19: Coronavirus disease 2019
Ct: Cycle threshold
GP: General practitioner
IQR: Interquartile range
MA: Medical assistant
NPV: Negative predictive value
OR: Odds ratio
POCT: Point of care test
PPV: Positive predictive value
(RT-)PCR: (Reverse transcription) polymerase chain reaction
SARS-CoV-2: Severe acute respiratory syndrome coronavirus type 2
SD: Standard deviation
