# Supplementary material for: Diagnostic accuracy and feasibility of a rapid SARS-CoV-2 antigen test in general practice – a prospective multicenter validation and implementation study
Source: BMC Prim Care. 2022 Jun 11;23:149. doi: 10.1186/s12875-022-01756-1 (PMC9187884; doi:10.1186/s12875-022-01756-1)
Supplement: Supplementary file 1 — Additional file 1. Questionnaire MA. [file 12875_2022_1756_MOESM1_ESM.pdf]

# Questionnaire MA

## 1. Organizational and logistical effort

1.1 How many people in total did you include in the study?

☐ 0 ☐ 1-24 ☐ 25-49
☐ 50-74 ☐ 75-100 ☐ >100

1.2 How many minutes in average did it take you to perform one rapid test? (Meaning only the actual working, not including the time spent waiting for the result to be read).

1.3 How many employees from the practice were involved in the immediate implementation of the rapid test? (Recruitment, patient information, performance of the test, documentation)

1.4 How well could the rapid test be integrated into the regular practice workflows? very bad ☐ ☐ ☐ ☐ ☐ ☐ very good

1.5 Were there factors that disrupted the workflow in your practice? If so, can you give examples?

## 2. Consequences for the treatment

2.1 Do you think that in general the use of tests in primary care practices can have a positive effect on the infection situation? do not agree ☐ ☐ ☐ ☐ ☐ ☐ totally agree

2.2 How useful do you consider the use of rapid tests in general practitioners' practices? not useful ☐ ☐ ☐ ☐ ☐ ☐ very useful

2.3 Do you think that the rapid test conducted by a doctor has advantages over the rapid test sold over-the-counter (self-test or lay test) and conducted by the patient himself?

☐ Ja ☐ No



MUSTER

## 2. Consequences for the treatment [Fortsetzung]

2.4 Can you give examples of this?

## 3. Satisfaction with the study

| | | | | | | | | | |
|---|---|---|---|---|---|---|---|---|---|
| 3.1 How would you rate the organization of the study in general? | very bad | ☐ | ☐ | ☐ | ☐ | ☐ | ☐ | very good |
| 3.2 How would you rate the support you received during the study? | very bad | ☐ | ☐ | ☐ | ☐ | ☐ | ☐ | very good |

3.3 What did you like/what did you not like about this study?

## 4. Personal and practice information

4.1 Gender

☐ male ☐ female ☐ diverse

4.2 Age in years

☐ <35 ☐ 35-44 ☐ 45-54
☐ 55-64 ☐ 65-74 ☐ >74

4.3 How many years have you been working in this profession?

☐ <5 ☐ 5-9 ☐ 10-14
☐ 15-19 ☐ 20-24 ☐ 25-29
☐ 30-34 ☐ 35-39 ☐ >39

4.4 Type of practice

☐ Individual practice ☐ Group practice ☐ Medical care center

4.5 Location of the practice of your primary occupation

☐ rural (<5.000 inhabitants) ☐ Small town (5,000-<20,000 inhabitants) ☐ Medium size city (20,000-100,000 inhabitants)
☐ Big city (>100,000 inhabitants)



MUSTER
