# Supplementary material for: Diagnostic accuracy and feasibility of a rapid SARS-CoV-2 antigen test in general practice – a prospective multicenter validation and implementation study
Source: BMC Prim Care. 2022 Jun 11;23:149. doi: 10.1186/s12875-022-01756-1 (PMC9187884; doi:10.1186/s12875-022-01756-1)
Supplement: Supplementary file 2 — Additional file 2. Questionnaire GP. [file 12875_2022_1756_MOESM2_ESM.pdf]

Questionnaire GP

1. Organizational and logistical effort

1.1 How many people in total did you include in the study?

☐ 0 ☐ 1-24 ☐ 25-49
☐ 50-74 ☐ 75-100 ☐ >100

1.2 How many minutes in average did it take you to perform one rapid test? (Meaning only the actual working, not including the time spent waiting for the result to be read).

1.3 How many employees from the practice were involved in the immediate implementation of the rapid test? (Recruitment, patient information, performance of the test, documentation)

1.4 How would you rate the feasibility of the rapid test overall? very simple ☐ ☐ ☐ ☐ ☐ ☐ very complicated

1.5 What could be changed to make the rapid test easier to perform?

2. Consequences for the treatment

2.1 In how many of 10 patients did the rapid test help you decide on further medical treatment?

☐ 0 ☐ 1 ☐ 2
☐ 3 ☐ 4 ☐ 5
☐ 6 ☐ 7 ☐ 8
☐ 9 ☐ 10

2.2 Can you give examples of this?

2.3 Do you think that in general the use of tests in primary care practices can have a positive effect on the infection situation? do not agree ☐ ☐ ☐ ☐ ☐ ☐ totally agree

2.4 How useful do you consider the use of rapid tests in general practitioners' practices? not useful ☐ ☐ ☐ ☐ ☐ ☐ very useful

MUSTER

2. Consequences for the treatment [Fortsetzung]

2.5 Out of 10 patients with suspected COVID-19 infection, in how many cases will you use the rapid test in the future?

☐ 0 ☐ 1 ☐ 2
☐ 3 ☐ 4 ☐ 5
☐ 6 ☐ 7 ☐ 8
☐ 9 ☐ 10

2.6 What would be an appropriate amount for the billing of the rapid test? (Amount in Euro)

2.7 Are there specific situations in which you would prefer to use the rapid test to diagnose SARS-CoV-2 infection? ☐ Yes ☐ No

2.8 Can you give examples of this?

2.9 Do you think that the rapid test conducted by a doctor has advantages over the rapid tests sold over-the-counter (self-test or lay test) and conducted by the patient himself? ☐ Yes ☐ No

2.10 Can you give examples of this?

3. Satisfaction with the study

3.1	How would you rate the organization of the study in general?	very bad ☐ ☐ ☐ ☐ ☐ ☐ very good
3.2	How would you rate the support you received during the study?	very bad ☐ ☐ ☐ ☐ ☐ ☐ very good

MUSTER

3. Satisfaction with the study [Fortsetzung]

3.3 What did you like/what did you not like about this study?

4. Personal and practice information

4.1 Gender

☐ male ☐ female ☐ diverse

4.2 Age in years

☐ <35 ☐ 35-44 ☐ 45-54
☐ 55-64 ☐ 65-74 ☐ >74

4.3 General practitioner professional experience in years

☐ <5 ☐ 5-9 ☐ 10-14
☐ 15-19 ☐ 20-24 ☐ 25-29
☐ 30-34 ☐ 35-39 ☐ >39

4.4 Type of practice

☐ Individual practice ☐ Group practice ☐ Medical care center

4.5 Location of the practice of your primary occupation

☐ rural (<5.000 Inhabitants) ☐ Small town (5,000-<20,000 inhabitants) ☐ Medium size city (20,000-100,000 inhabitants)
☐ Big city (>100,000 inhabitants)

MUSTER
