# Supplementary material for: Diagnostic accuracy and feasibility of a rapid SARS-CoV-2 antigen test in general practice – a prospective multicenter validation and implementation study
Source: BMC Prim Care. 2022 Jun 11;23:149. doi: 10.1186/s12875-022-01756-1 (PMC9187884; doi:10.1186/s12875-022-01756-1)
Supplement: Supplementary file 3 — Additional file 3. Attachement Table 1 Baseline characteristics of MAs ans GPs who followed the survey. [file 12875_2022_1756_MOESM3_ESM.pdf]

Attachment table 1: Baseline characteristics of MAs and GPs who followed the survey

	GP[a], n=40*		MA[a], n=39*	
	n	(%)	n	(%)
Sex				
Male	19	(51.4)	3	(7.9)
Female	18	(48.6)	35	(92.1)
Age				
<35	2	(5.4)	10	(27.8)
35-54	24	(64.9)	21	(58.3)
55-74	11	(29.7)	5	(13.9)
Work experience in years				
<5	6	(16.7)	10	(27)
5-9	4	(11.1)	3	(8.1)
10-19	11	(30.6)	12	(32.4)
>20	15	(41.7)	12	(32.4)
Type of medical practice				
Community practice	22	(61.1)	23	(62.2)
Single practice	14	(38.9)	14	(37.8)
Location of the practice				
Rural (<5.000 Inhabitants)	13	(36.1)	11	(29.7)
Small town (5.000-20.000 Inhabitants)	6	(16.7)	7	(18.9)
Medium size city (20.000-100.000 Inhabitants)	4	(11.1)	4	(10.8)
Large city (>100.000 Inhabitants)	13	(36.1)	15	(40.5)

*missing values range between 1 and 4

[a]GP = general practitioner, MA = medical assistant
